# Conflicts of interest and the future of medicine: the US, France, and Japan

**Published:** 2012-01-30

**Authors:** Thomas Kostera

**Affiliations:** MES Dipl.-Verw.Wiss. Institute for European Studies/CEVIPOL Université libre de Bruxelles (ULB), Avenue Roosevelt 39, 1050 Bruxelles, Belgium Phone: +32 (0)2 650 45 48 E-mail: Thomas.Kostera@ulb.ac.be

In his book Marc Rodwin, Professor of Law at Suffolk University, analyses the regulation of medical interests. He looks more precisely at doctors’ conflicts of interest that can have an influence on their therapeutic choices which are not necessarily in the patients’ best interest. While society and regulators usually expect doctors to be objective in their therapeutic choices, regulating (or the lack thereof) entrepreneurship of private practitioners, their ownership of medical facilities, their type of employment (private, public or mixed), and forms of remuneration for medical services can create incentives for preferring one medical treatment over another. The initial chapter of the book illustrates these choices by presenting fictional patients from France, the US and Japan who share the same diagnosis, but receive a variety of treatments depending on the economic and regulatory incentive structure of medical practice.

The book’s main research question is a normative one, namely how regulation can minimize conflicts of interest between the patients’ interest and physicians’ entrepreneurial goals. On the basis of a political economic perspective, the book sets out to analyse the interplay between several variables: medical associations’ oversight and medical self-regulation, market competition mechanisms, insurance companies’ influence over medical practice, and the state’s practice of regulation. This analytical framework is developed in chapter 1.

Chapters 2 and 3 deal with France. The development of the relationship between the organized medical profession, insurance companies and the role of the state are traced back from the medieval times onwards in chapter 2. The last section of the chapter also looks at the influence of European law. Chapter 3 analyses how France aims at avoiding conflicts of interest. The author shows the unusual strength of the French Medical Association and how certain relationships between the pharmaceutical industry and doctors are still tolerated. Rodwin concludes that France only shows limited success in dealing with doctors’ conflicts of interest.

Chapters 4–7 form the core of the book and deal with the US. Rodwin distinguishes four phases of the development of the medical economy showing a high variation in tackling medical conflicts of interest. Chapter 4 covers the period before 1950, chapter 5 the period until 1980 and chapter 6 the logic of medical markets from the 1980s onwards. Chapter 7 deals with the ways in which the US cope with conflicts of interests today. The author shows how insurance companies have come to set incentives to reduce medical services and thus create conflicts of interest. Also, the market orientation of the American healthcare system has reinforced ties between physicians and the pharmaceutical industry. Rodwin recommends federal regulation of medical care and health insurance, in order to develop a coherent approach to coping with conflicts of interest.

The following chapters focus on Japan. Chapter 8 depicts the historical development of Japan’s medicine and chapter 9 analyses how Japan copes with conflicts of interest. Rodwin exposes the coexistent roles of Japanese doctors as private and hospital practitioners leading to a situation in which Japanese patients stay longer in hospitals than in other nations and also receive more drugs for medical treatment.

Chapter 10 (‘Reforms’) deals with the implications of the previous findings for regulation. Neither market competition nor pure public employment of physicians alone does necessarily mitigate conflicts of interests of doctors. Hence, both should coexist. Some of the other suggested solutions are strict regulation of entrepreneurship of private practitioners, of ties between doctors and the pharmaceutical industry, and avoiding intervention of insurance companies in medical standard setting.

Chapter 11 is a more sociology-inspired chapter dealing with the concept of professionalism of physicians and its role to play in reducing conflicts of interest. Rodwin argues that the state, doctors and market mechanisms alike should have authority to regulate conflicts of interests, thus effectively providing for the possibility of ‘checks and balances’ between them.

The book provides overall a very detailed analysis of the historical and structural sources for conflicts of interest in the three countries presented. The chapter on professionalism complements the political economic perspective and avoids an overly functionalist view of coping with conflicts of interests. The detailed analysis shows that the state and insurance funds are also no ‘neutral’ actors and develops therefore to the convincing conclusion that conflicts of interest are best dealt with by a mix of market-driven, professional and public regulation. The detailedness of some chapters complicates however the readability and leaves the reader with the question if the same conclusions and recommendations could not have been developed with a more structured presentation of some developments.

While the ‘patients’ interest’ plays a key role for analysis, the book falls short of defining what the patients’ interests would be* from a regulatory perspective*. These interests are not necessarily congruent with the individual patient’s interest of receiving the best medical care. From a regulator’s perspective patients are one interest group among others, even if they are certainly one of the most important groups given their role as future electors. Yet, their interest has to be reconciled with other legitimate interests. Since the medical profession is the main object of interest for the book, it would also be desirable to inquire about the belief structures of physicians about what their own and what patients’ interests are. Using regulatory incentive structures alone does not necessarily explain why certain doctors themselves criticize the ties between the pharmaceutical industry and their profession, even though the same regulatory incentive structures apply.

Thanks to its comprehensive analysis of the three countries and their different regulatory frameworks this book is not only useful for legal or economic scholars/experts who are interested in dealing with conflicts of interest, but also for those who would like to study the healthcare systems of France, Japan and the USA. It is also useful as a starting point for sociologists and political scientists for studying the role of the medical profession.

